# Temporal Activity Patterns of Sympatric Species in the Temperate Coniferous Forests of the Eastern Qinghai-Tibet Plateau

**DOI:** 10.3390/ani13071129

**Published:** 2023-03-23

**Authors:** Jia Jia, Yun Fang, Xinhai Li, Kai Song, Wendong Xie, Changli Bu, Yuehua Sun

**Affiliations:** 1 Key Laboratory of Animal Ecology and Conservation Biology, Institute of Zoology, Chinese Academy of Sciences, Beijing 100101, China; 2 University of Chinese Academy of Sciences, Beijing 100049, China

**Keywords:** temporal overlap, activity rhythm, coexistence, camera trap, relative abundance index

## Abstract

**Simple Summary:**

This study provides information on the temporal relationships of ground-dwelling birds and mammals in the temperate coniferous forests on the eastern Qinghai-Tibet Plateau based on infrared camera technology and reveals the coexistence pattern of the dominant species in this ecosystem for potential community interactions. We explored daily activity patterns and seasonal variations in temporal niche overlap for eight sympatric species. We found partitioned temporal activity among the studied species and their temporal niche overlap differed between the cold and warm seasons, indicating potential competition intensity related to climate and resource conditions. The goals of this study were to explore the temporal niche coexistence of dominant species in coniferous forests by calculating and comparing the daily activity rhythms and seasonal rhythms, and to compare the competitive pressure of species pairs in similar habitats inside and outside the reserve, so as to provide information for understanding the species in the Qinghai-Tibet Plateau. Our study aimed to set a baseline for understanding the mechanisms of ecological interactions among sympatric species. Furthermore, the results of this work could be used to assess the conservation status of sympatric animals in the study area, which could provide important guiding significance for future protection and management of the studied reserve.

**Abstract:**

Temporal niche partitioning is an important strategy for sympatric species or populations when utilizing limited resources while minimizing competition. Different resource availability across seasons may also influence the intensity of competition, resulting in a varied temporal niche partitioning pattern between species. These competitive interactions are important drivers for the formation of biodiversity patterns and species coexistence on the eastern Qinghai-Tibet Plateau. To clarify these interspecies relationships among sympatric species, we carried out a camera trap survey from 2017 to 2020. We deployed 60 camera traps in the temperate coniferous forests of the eastern Qinghai-Tibet Plateau. We analyzed the daily activity patterns of birds and mammals to reveal the temporal niches and seasonal relationships among the species-specific activity rhythms. The results are summarized as follows: (1) Eight major species, including mammals and birds, have different temporal peak activity rhythms to reduce intense competition for resources. (2) The activity rhythm of a species varies seasonally, and the competition among species is more intense in the warm season than in the cold season. (3) Among 15 pairs of competitor species, seven pairs had significantly different coefficients, with higher winter values than summer values, perhaps due to the abundance of resources in summer and the scarcity of resources in winter causing intensified competition. Among the predators and prey, the summertime coefficients were higher than those in winter, perhaps due to the need to replenish energy during the summer breeding season. The main purpose of animals in winter is to survive the harsh environment. Our results provide important information on temporal and interspecies relationships and contribute to a better understanding of species-coexistence mechanisms.

## 1. Introduction

Interspecific interactions are a classical and vivid research topic [1], and the competitive exclusion principle states that ecologically similar species cannot coexist. Thus, species that coexist are thought to be segregated in a horizontal, vertical or temporal dimension, and multiple sympatric species can partition their niches according to major factors, including food resources [2], space [3], and time [4], to reduce competition and achieve coexistence. Species living in similar environments are mainly involved in predator-prey and related relationships. Predators are known to synchronize their predatory activities with the activity periods of their primary prey and co-predators [5]. Some studies have indicated that temporal activities of predators are likely driven largely by the circadian activities of their main prey species, especially with regard to carnivores [6]. Predators can maximize the pay-off for hunting by doing so when prey are most vulnerable to attack, and prey can improve their survival by reducing their temporal niche overlaps with predators [7].

For ecologically similar relative species, coexistence mechanisms are another important issue [8,9]. Species must segregate along one or more dimensions within their ecological niches and differ in their use of limited resources in order to coexist [4]. Niche partitioning includes resource partitioning and temporal partitioning; the closer the relationship between two species is, the more competitive the species niche is. Darwin viewed organic evolution as primarily a combination of variation and the struggle for survival, primarily as a struggle for competition [10]. Evolution is an effective way to avoid fierce competition and the most efficient way for species to coexist. Comparative studies of sympatric species are essential for understanding those species’ behavioral and ecological adaptations [11]. Interspecific competition plays an important roles in shaping communities by affecting the ability of component species to access limited resources [12].

The temporal segregation when utilizing habitats and resources can be reflected by the temporal activity of species. These patterns are highly variable across seasons within the same species [13]. Thus, considering how temporal activity varies seasonally may better explain how sympatric species avoid competition in different climatic conditions [14]. However, previous study of interspecies relationships mainly involved analyses of the temporal avoidance by predators and prey [5,6,15,16] and resource allocation among closely related species [17,18,19,20]. Relatively few studies have been carried out on the sympatric coexistence of birds, involving only ground-dwelling pheasant birds and the competitive relationship between them and mammals [21]. In this study, utilizing non-invasive monitoring technology via infrared cameras, the temporal relationships among different bird and mammal species in the study area were discussed to provide reference materials for species intermediate relationships.

Given the technological and statistical advances in the application potential of camera trapping [21], camera traps are widely used in ecology and conservation for investigating species distributions and biodiversity inventorying [22,23]; such cameras have become a cost-effective tool providing researchers with a large amount of data [24]. Past abundance and spatiotemporal distribution studies have focused mainly on large predators and ungulates [7,20,23], while biodiversity monitoring and regional inventories have focused mainly on ground-dwelling bird species [25,26,27,28]. Infrared camera pictures provide temporal and spatial information for spatiotemporal behavior analyses [29], and the diurnal activity rhythm and temporal overlap of species have become popular research topics [7,30]. However, species’ behaviors and interactions and their associated consequences with regard to community composition have been examined less often. Temporal camera-trap data offer the opportunity to address unresolved questions regarding species ecology and community interactions, and the use of time as a resource by species can provide valuable information about their ecological niches [31]. Numerous studies have observed temporal niche partitioning as an important strategy enabling the coexistence of ecologically similar species [4,17,32]. Animal activity patterns evolve via processes of natural selection [33], but behavioral plasticity may allow flexible changes to activity patterns in response to environmental stimuli, such as during periods of higher predation risk [34]. Ultimately, understanding particular biological processes and different times of the same species, how various species meet their needs for food acquisition, thermoregulation, and danger avoidance can be achieved by observing the activity patterns of various species. Further, comparing their activity patterns is crucial to comprehending the mechanisms governing species interactions [35,36].

Wildlife surveys are the basis of biodiversity protection, as they provide basic data and technical support for biodiversity conservation and sustainable use and the implementation of the Convention on Biological Diversity (https://www.cbd.int/ (accessed on 2 February 2023)). In this study, camera traps were also placed in similar habitats outside the protected area to form a control group. By comparing the overlap coefficient of the same species pairs, the competitive pressure of species in and outside the protected area was evaluated, which provided a scientific basis for the subsequent planning and work in the protected area.

## 2. Materials and Methods

### 2.1. Study Area

The study area provides a typical temperate coniferous forest habitat on the eastern Qinghai-Tibet Plateau. The average annual temperature is 5.1–6.0 °C, with a maximum temperature of 34 °C and a minimum temperature of −27.1 °C. The site has a continental climate. The annual precipitation is approximately 650 mm, and rainfall is concentrated from June to August in summer. The area is covered by snow from November of each year to April of the next year [37]. Due to the high elevation and relatively little human disturbance, this area has become a breeding ground for many rare wild animals, such as Chinese grouse (*Bonasa sewerzowi*) and Sichuan wood owl (*Strix davidi*), both of which are endemic species. The evergreen coniferous forest also provides a wintering place and food for some species with weak migration abilities, with 745 species of seed plants and 10 species of plants listed as national key protected species in China [37]. The dominant vegetation includes spruces, firs, willows and birches. The seasons can be divided into two periods according to climatic data from the weather station of the study area: April to September is the warm season, and October of each year to March of the next year is the cold season.

### 2.2. Data Collection

**Figure 1:**
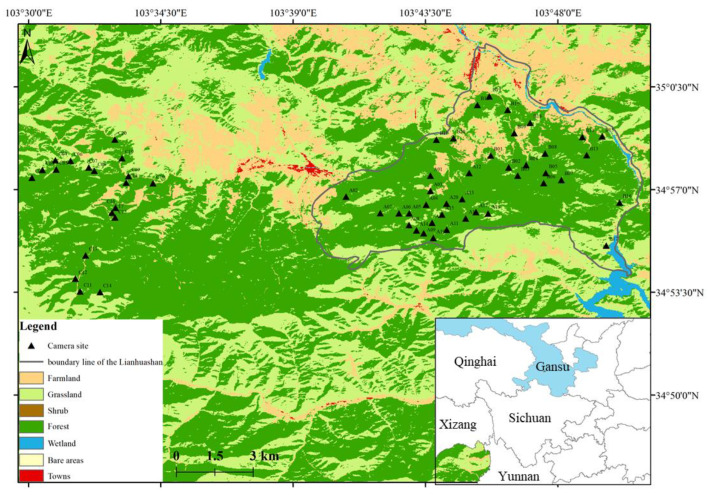
Camera trap locations (black triangles) distributed in the temperate coniferous forests of the eastern Qinghai-Tibet Plateau, China.

Sixty infrared cameras (model LTl-6218, Shenzhen, China) were deployed from September 2017 to December 2020 in the study area. The east side included forty camera traps placed in Lianhuashan National Nature Reserve in Gansu Province, while twenty cameras on the west side were the control group, placed in similar habitats outside the reserve (Figure 1, Appendix A). The spacing between camera sites was about 500 m, and the cameras were placed within a 1 × 1 km grid, based on tracks and sign knowledge of local guides to increase the probability of capturing photos of wildlife [38]. The camera traps were fixed mainly at a position 0.5–0.8 m from the forest floor, and the lens of each camera was oriented in a direction parallel to the ground, facing away from dense vegetation. We programmed the cameras in photo and video patterns, with 3 images and one 15 s video captured per trigger event, and the interval was set at 3 s. We checked the memory cards and batteries every 3 months and adjusted the cameras for broad vision to record additional species [39].

The locations of the infrared camera traps were drawn in ArcGIS 10.7 (ESRI), and the vegetation type composing the background was derived from the global 30 m land cover layer in 2020 [40].

### 2.3. Data Analysis

Consecutive detections of the same species at a camera-trap site within 30 min were treated as a single sample in order to reduce duplicate counts of the same individual [4]. The calculated metrics included the number of trap nights (or camera days) spent by one camera at the *i*-th location (TN); the number of animal passes/registrations (TE–trap events) at the *i*-th location; the number of independent animal registrations; and the relative abundance index (RAI or TS–trap success), which was calculated using the formula [23] as follows:

$$\mathrm{RAI}=(\sum_{i=1}TE_{i}/\sum_{i=1}TN_{i})\times 100$$

We defined the dawn (05:00–07:00) and dusk (17:00–19:00) time periods as one-hour periods before and after sunrise/sunset, respectively, and the intervening periods as day (07:00–17:00) and night (19:00–05:00) [41]. In accordance with Schaik and Griffiths (1996) [42], we divided observations into diurnal (<15% of the observations obtained at night), nocturnal (>85% of the observations obtained at night), mostly diurnal (15–35% of the observations obtained at night), mostly nocturnal (65–85% of the observations obtained at night), crepuscular (50% of the observations obtained during the crepuscular period), and cathemeral (species active both day and night) [35,43,44].

To estimate the animal activity pattern, we used the approach developed by Ridout and Linkie [22] to estimate the activity patterns of each species in each season using kernel density. The temporal overlap of every species pair was calculated using an overlapping coefficient with the R package "overlap" [45] in R 4.1.1. The densityPlot function was used to draw a single plot of the kernel density of species, and the overlapEst function was used to estimate the overlapping coefficient (Δ ranged from 0, indicating no overlap, to 1, indicating total overlap). Of the several estimator Δ metrics, Δ_4_ was recommended for sample sizes larger than 75, and the temporal overlaps of activity patterns were ranked by considering high overlap when ∆ > 0.75, moderate overlap when 0.50 < ∆ < 0.75, and low overlap when ∆ < 0.50 [16]. Function compareCkern () of package “activity” [46] was used to test whether two sets of circular observations came from the same distribution, reps = 10,000. The Wald test using the function compareAct () was used to estimate the significance of pairwise comparisons between overall activity levels.

Activity data presented a circular distribution, so we compared the distributions of several activity pattern samples among seasons using the nonparametric Watson–Wheeler test of homogeneity of means with the R package “circular” [47,48]. This test indicates whether there is a significant difference between circular distributions and has been used to analyze data corresponding to 24 h activity patterns [49,50]. Bootstrap analysis to estimate the confidence intervals (CI) of the coefficient of overlapping generated 10,000 smoothed bootstraps for pairwise comparisons.

We followed the same procedures to test activity overlaps between predators and prey according to season. We identified which species exhibited significantly low overlaps with the study fields and those that showed significantly high overlap patterns, which we defined as below the 5th percentile and above the 95th percentile of overlap across all candidate prey species, respectively. All statistical analyses were performed in R software [51].

## 3. Results

During the 4-year camera-trapping period, with a total of 49,190 camera days, we obtained 12,959 images of wild animals from 60 locations. We collected 4879 independent capture events with eight terrestrial species. The number of events included 3377 records of the eastern roe deer *Capreolus pygargus* (warm season, 1906; cold season, 1471), 428 records of the blue-eared pheasant *Crossoptilon auritum* (warm season, 211; cold season, 217), 266 records of the tolai hare *Lepus tolai* (warm season, 116; cold season, 150), 184 records of the wild boar *Sus scrofa* (warm season, 91; cold season, 93), 174 records of the Asian badger *Meles leucurus* (warm season, 139; cold season, 35), 155 records of the common pheasant *Phasianus colchicus* (warm season, 54; cold season, 101), 148 records of the red fox (warm season, 65; cold season, 83), and 147 records of the blood pheasant *Ithaginis cruentus* (warm season, 72; cold season, 75). The maximum values of the relative abundance index were noted for the eastern roe deer (RAI = 6.87) and blue-eared pheasant (RAI = 1.03). (Table 1).

### 3.1. Daily Activity Rhythms of the Eight Dominant Species

**Figure 2:**
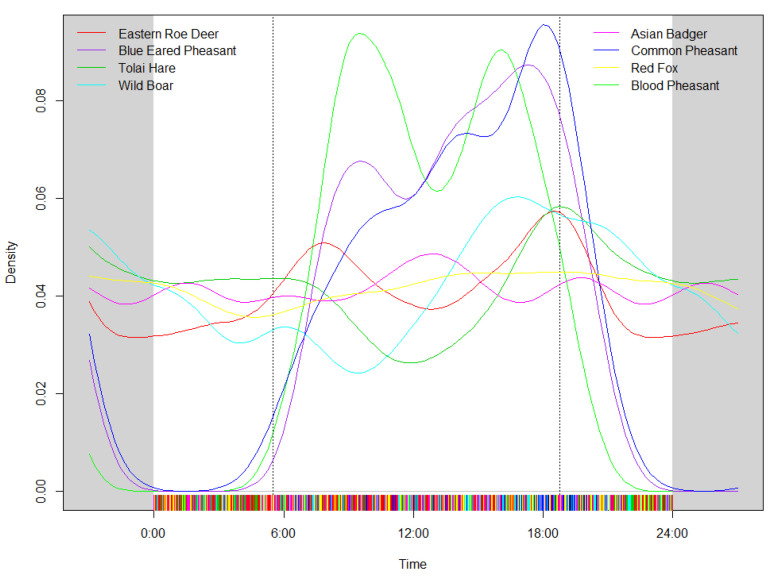
Kernel densities of daily activity of eight animal species (solid line) according to the data from camera traps. Two vertical lines represent the average time of sunrise and sunset, on the eastern Qinghai-Tibet Plateau in 2017–2020.

Considering the day as a period of 24 h, it is possible to present activity patterns in the form of density plots, and eight species analyzed herein varied greatly with regard to how they utilized the diel period (Figure 2; Table 1). Birds were obviously diurnal. The blue-eared pheasant and blood pheasant exhibited a bimodal activity type, but the peak activity of blood pheasant at dawn was higher than that at dusk, while the distribution of blue-eared pheasant activities was the opposite. The common pheasant had a unimodal activity type with one peak at dusk. Mammals were both diurnal and nocturnal. The eastern roe deer had a bimodal activity type and strong activity behavior at both dawn and dusk. Some mammal species, such as the wild boar, tolai hare, and Asian badger, had unimodal activity types with one peak; these species had activity peaks before 18:00, after 18:00 and at 12:00, respectively. The red fox was more active during both day and night than the other captured species. Comparing the activity time proportions of these eight dominant species, we found that the wild boar and Asian badger had the highest activity proportions at dawn, the three pheasant species had the highest proportions in the daytime and at dusk, and the three mammals (the tolai hare, wild boar, and red fox) had the highest proportions at night.

### 3.2. Daily Activity Patterns during the Warm Season and Cold Seasons of the Year

**Figure 3:**
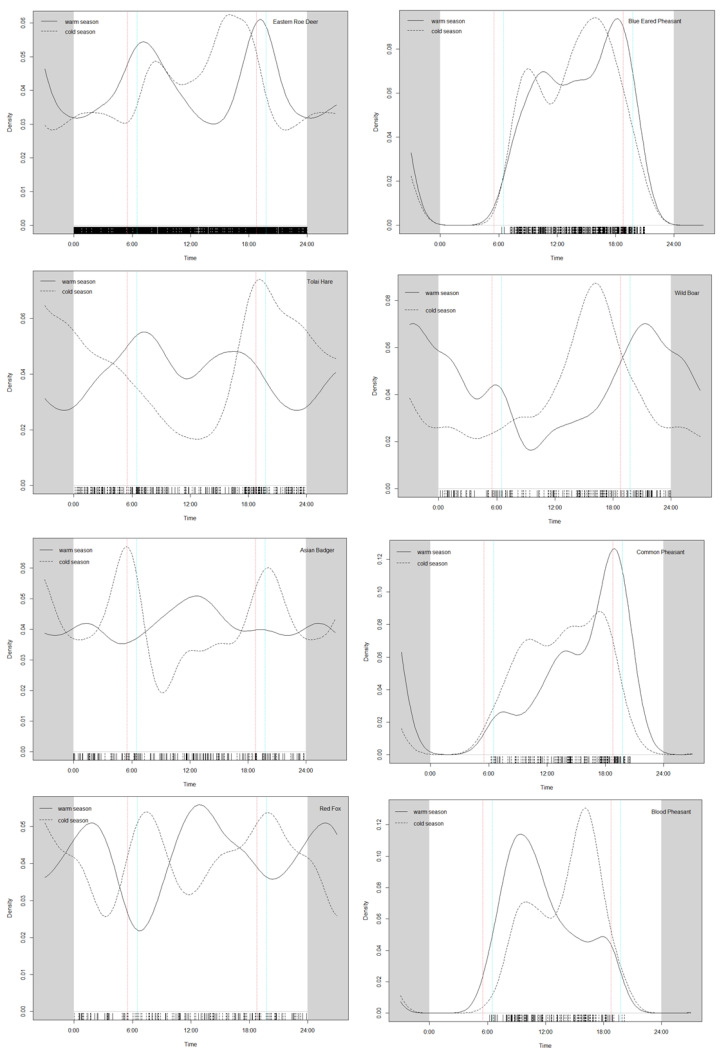
Black dotted and solid lines represent the seasonal (warm season and cold season) activity patterns of the eight dominant species, the red line indicates the time of sunrise (**left**) and sunset (**right**) in the warm season, and the blue line indicates the time of sunrise (**left**) and sunset (**right**) in the cold season, on the eastern Qinghai-Tibet Plateau (2017–2020).

The activity rhythms of species can vary between the cold season and warm season each year (Figure 3). Among the eight analyzed species, we found an extremely significant difference (*p* < 0.01) in the activity rhythms of the eastern roe deer (*p* < 0.05), tolai hare (*p* < 0.05), wild boar (*p* < 0.05) and blood pheasant (*p* < 0.05) between these two seasons. The first peak of the eastern roe deer in the cold season moved backwards, and the second peak moved forwards. The tolai hare had two activity peaks in the warm season and only one peak in the cold season. The first peak of the wild boar in the cold season moved backwards, as did that of the eastern roe deer. The activity peak of the blood pheasant in the warm season was close to dawn, while in the cold season, it was close to dusk. The activity rhythm of the common pheasant in the cold season was highest at dusk.

### 3.3. Diel Temporal Overlaps of the Dominant Species

We calculated the overlapping coefficients of the daily activity rhythms of the dominant species (Table 2). Among the predator-prey pairs, the red fox and tolai hare expressed a high level of temporal overlap (Δ_4_ = 0.89, *p* = 0.18). The remaining nine pairs moderately overlapped, and showed significant differences (*p* < 0.05). Regarding temporal overlaps among competitor species, 11 pairs showed high overlaps, while four pairs showed moderate overlaps. Significant differences were observed in seven pairs of highly overlapping species, including the wild boar, which highly overlapped with the eastern roe deer (95% CI: 0.8–0.92, *p* = 0.01), tolai hare (95% CI: 0.8–0.93, *p* = 0.03) and Asian badger (95% CI: 0.78–0.93, *p* = 0.04). The blood pheasant highly overlapped with the blue-eared pheasant (95% CI: 0.79–0.92, 0.02) and common pheasant (95% CI: 0.7–0.88, *p* = 0.01). The tolai hare highly overlapped with the eastern roe deer (95% CI: 0.84–0.93, *p* = 0.01) and Asian badger (95% CI: 0.8–0.93, *p* = 0.07). Significant differences were observed in four pairs of moderately overlapping species: the tolai hare moderately overlapped with the three pheasant species, the common pheasant (95% CI: 0.53–0.68, *p* < 0.01), blue-eared pheasant (95% CI: 0.53–0.63, *p* < 0.01) and blood pheasant (95% CI: 0.46–0.6, *p* < 0.01). The blood pheasant and eastern roe deer (95% CI: 0.57–0.68, *p* < 0.01) also moderately overlapped.

Among the predator-prey pairs (Table 3), the daily activity overlapping coefficients of the wild boar and three pheasant species were higher in winter than in summer. Following interspecific temporal partitioning (above the 95th percentile of overlapping coefficient values), the overlaps between the badgers and three pheasant species were found to be higher in summer than in winter; these species highly overlapped during the warm season, but overlapped less in the cold season. In summer and winter, the red fox and three pheasant species overlapped moderately, and the red fox and tolai hare overlapped highly. Among competitor species (Table 4), seven pairs had coefficients that were significantly different between winter and summer, with higher values in winter than in summer; in addition, the roe deer and blue-eared pheasant moderately overlapped, and the wild boar and roe deer highly overlapped.

Regarding the activity overlap differences of the predator and prey species between the warm season and cold season, the summertime coefficients were higher than those in winter. The tolai hare and three pheasant species overlapped moderately in the warm season and overlapped slightly in the cold season, while the wild boar and badger overlapped highly in the warm season and overlapped moderately in the cold season.

## 4. Discussion

### 4.1. Daily Activity Rhythms of Dominant Species

The temporal activity patterns of the studied species suggest daily time utilization niche partitioning and seasonal variation [52]. These daily and seasonal temporal patterns revealed the niche-partitioning mechanisms among sympatric species as well as the species coexistence mechanisms and community composition. The daily activity of species is related to the daily activity rhythm of their prey [6], while intraspecific competitive relationships among species can also affect their daily activity rhythms [53]. In addition, humans can produce habitat modifications, mainly through disturbances [54] (e.g., poaching), that may change the daily activities of species.

Many studies have shown that species are active at different times of day to avoid competition. The separation in carnivore activity peaks reduces the chances of interspecific killing and contributes to interspecific coexistence [45]. Moderate temporal partitioning, such as the coexistence of the largest carnivorous marsupial, the Tasmanian devil (*Sarcophilus harrisii*), and the smaller spotted-tail quoll (*Dasyurus maculatus*), involves the utilization of similar habitats and consumption of similar prey species [9].

Our results showed that mammals were active at night, while pheasants were active during the day. Some studies have shown that mammals that are active at night may do so to avoid human activities [55,56]. The use of different peak activity times is a way for closely related species to avoid temporal niche competition, and fixed activity rhythms are associated with predators and environmental factors [7,57]. The activity levels of wild boars were the same in the morning and evening and peaked at 16:00, while the activity of wild boars at night was multiple times greater than that in the daytime. The Asian badger was more active at dawn than at dusk and in the daytime than at night. The red fox activity peaked at 12 a.m. Tolai hare were more active at dusk than at dawn and more active at night than during the day; their activity peak occurred at 18:00. The eastern roe deer was the representative ungulate, and its activity peak occurred at 18:00. The evening activity peaks of the blood pheasant, blue-eared pheasant and common pheasant were at 16:00, 17:00 and 18:00, respectively, while their morning activity peaks were at 8:00, 10:00 and 11:00, respectively. It is possible that the body size and nutritional requirements of these three pheasant species were the main factors affecting their activity rhythms, but further proof is needed [21].

### 4.2. Daily Activity Patterns during the Warm Season and Cold Season of the Year

Seasonality has a compounding effect on animal physiology, movement ecology, foraging strategy and survival in response to changes in temperature, precipitation, food availability, etc. [20,58,59]. With the availability of resources and variation of climate conditions [13], seasonal variation in temporal activity patterns may reflect the intensity of competition among sympatric species [4].

It is, therefore, meaningful to study animal activity rhythms with respect to distinct seasons, and in this study, the activity rhythms of four of the target species exhibited seasonal differences to varying degrees. The red fox preys on the tolai hare. In winter, the tolai hare was active from 17:00 to 20:00, while in summer, it was active from 6:00 to 14:00 and from 15:00 to 19:00. There was no evidence of avoidance behavior by the tolai hare toward the red fox. These seasonal differences may thus be due to the greater energy required for summertime reproduction than wintertime reproduction. European brown hares can shift their temporal activity patterns when terrestrial predators are present [7]. The eastern roe deer and wild boar lack predators and are competitors. The wild boar activity peaked in summer at 21:00, while the activity of this species in winter moved forward to 16:00. The eastern roe deer was bimodal in both seasons, with the wintertime activity peak moving to daytime. This may have been due to the low temperatures in winter causing the species to choose relatively warm times to reduce physical consumption. No avoidance behavior caused by competition was observed in either of these two species.

The activity peak of blood pheasant also moved with decreasing temperatures, with the morning peak being delayed and the evening peak advancing. In winter, the activity levels increased significantly from 15:00 to 16:00, possibly due to the cold winter temperatures and the requirement of this species to consume more energy over the long nights, resulting in the need to eat more food. 

### 4.3. Activity Overlap of the Dominant Species

The red fox preys on the tolai hare. The time rhythm overlap was high between these two species (Δ_4_ = 0.89, *p* = 0.18), and the overlapping coefficient in summer (Δ_4_ = 0.81, *p* = 0.03) was higher than that in winter (Δ_4_ = 0.76, *p* = 0.01); this result was related to the increased activity levels of the tolai hare in summer. The wild boar and eastern roe deer have a competitive relationship. Due to their lack of predators, their activities highly overlap in both summer and winter, indicating that these two species have intense competition for resources and require top predators to improve the local food chain. The wild boar, tolai hare and Asian badger highly overlap in summer and moderately overlap in winter. The tolai hare activity frequency decreased in winter, while the Asian badger exhibited a hibernation habit, thus producing a seasonal temporal rhythm. The three pheasant species highly overlapped throughout the year, including moderate overlaps identified between the blue-eared pheasant and blood pheasant and between the blood pheasant and common pheasant; some highly overlapping patterns were observed in both summer and winter due to the breeding season of these species occurring in summer. The overlapping coefficients of the pheasant species were small because of their different habitat types, though the overlapping coefficients were relatively high in winter because of the clustering activities adopted by these species to feed and avoid natural enemies.

The overlapping coefficients of competitors differed significantly in seven species pairs between the two seasons, and the overlapping coefficients of the activity rhythms of predator-and-prey pairs were higher in summer than in winter. Competition is fierce in summer, but in winter, it is more important for species to replenish their food resources and survive the cold season, thus causing both predation and energy consumption to be reduced.

We also investigated the temporal rhythms of the dominant species inside and outside a nature reserve, with n > 30 for six pairs of species. The results showed that the overlap rates of species outside the reserve were higher and the competition was more intense than those inside the reserve (Table 5), potentially because serious habitat destruction outside the reserve has resulted in a decrease in the area of suitable habitats for these species and an increase in resource competition among the species [55,58].

## 5. Conclusions

This study provided information on the temporal relationships of ground-dwelling birds and mammals in the temperate coniferous forests on the eastern Qinghai-Tibet Plateau based on infrared camera technology and revealed the coexistence pattern of the dominant species in this ecosystem for potential community interactions. We explored daily activity patterns and seasonal variation in temporal niche overlap for eight sympatric species. We found partitioned temporal activity among the studied species and different temporal niche overlap between the cold and warm seasons, indicating potential competition intensity related to climate and resource conditions. Our study aimed to set a baseline for understanding the mechanisms of ecological interactions among sympatric species. Furthermore, the results of this work could be used to assess the conservation status of the sympatric animals in the study area, which could provide important guiding significance for future protection and management of the studied reserve.

## Figures and Tables

**Table 1:** Percentage of independent events of eight species of animals in four different time periods.

Species	RAI	Dawn (05:00–07:00)	Day (07:00–17:00)	Dusk (17:00–19:00)	Night (19:00–05:00)
Eastern Roe Deer	6.87	8.68	43.35	11.76	36.22
Blue Eared Pheasant	1.03	0.70	69.63	18.69	10.98
Tolai Hare	0.54	9.02	30.83	14.29	45.86
Common Pheasant	0.42	4.52	58.06	24.52	12.90
Wild Boar	0.37	9.78	39.13	9.78	41.30
Asian Badger	0.35	11.49	41.38	8.62	38.51
Blood Pheasant	0.32	4.08	80.95	11.56	3.40
Red Fox	0.3	8.78	42.57	8.11	40.54

**Table 2:** Coefficients of daily activity overlapping (Dhat4) and their confidence intervals (CI) for eight animal species on the eastern Qinghai-Tibet Plateau in 2017–2020.

Eastern Roe Deer	Eastern Roe Deer						
Blue Eared Pheasant	0.67 (0.64–0.7)	Blue Eared Pheasant					
Tolai Hare	0.89 (0.84–0.93)	0.58 (0.53–0.63)	Tolai Hare				
Common Pheasant	0.68 (0.62–0.74)	0.91 (0.85–0.95)	0.60 (0.53–0.68)	Common Pheasant			
Wild Boar	0.87 (0.8–0.92)	0.62 (0.55–0.69)	0.87 (0.8–0.93)	0.65 (0.57–0.73)	Wild Boar		
Asian Badger	0.90 (0.85–0.94)	0.62 (0.55–0.69)	0.87 (0.8–0.93)	0.65 (0.57–0.73)	0.86 (0.78–0.93)	Asian Badger	
Blood Pheasant	0.63 (0.57–0.68)	0.86 (0.79–0.92)	0.53 (0.46–0.6)	0.80 (0.7–0.88)	0.58 (0.5–0.66)	0.59 (0.51–0.67)	Blood Pheasant
Red Fox	0.91 (0.86–0.95)	0.63 (0.56–0.71)	0.89 (0.82–0.94)	0.66 (0.57–0.74)	0.90 (0.83–0.95)	0.95 (0.92–0.98)	0.60 (0.52–0.68)

**Table 3:** Coefficients of daily activity overlapping (Dhat4), and *p*-level (*p*) and their confidence intervals (CI) for predatory species on the eastern Qinghai-Tibet Plateau in 2017–2020.

		Warm Season			Cold Season		
Species1	Species2	Dhat4	CI	*p*	Dhat4	CI	*p*
Blue Eared Pheasant	Wild Boar	0.48	0.37–0.59	0	0.76	0.67–0.84	0
Blue Eared Pheasant	Asian Badger	0.66	0.59–0.75	0	0.49	0.31–0.67	0
Blue Eared Pheasant	Red Fox	0.63	0.48–0.74	0	0.61	0.49–0.72	0
Wild Boar	Blood Pheasant	0.45	0.36–0.58	0	0.71	0.61–0.8	0
Wild Boar	Common Pheasant	0.54	0.44–0.65	0	0.73	0.59–0.84	0
Blood Pheasant	Asian Badger	0.64	0.53–0.71	0	0.44	0.28–0.56	0
Blood Pheasant	Red Fox	0.6	0.48–0.68	0	0.53	0.38–0.66	0
Tolai Hare	Red Fox	0.81	0.7–0.9	0.03	0.76	0.63–0.85	0.01
Common Pheasant	Asian Badger	0.61	0.49–0.72	0	0.49	0.31–0.67	0
Common Pheasant	Red Fox	0.59	0.48–0.71	0	0.59	0.45–0.72	0

**Table 4:** Coefficients of daily activity overlapping (Dhat4), confidence intervals (CI) and *p*-level (*p*) for competitor species on the eastern Qinghai-Tibet Plateau in 2017–2020.

		Warm Season			Cold Season		
Species1	Species2	Dhat4	CI	*p*	Dhat4	CI	*p*
Eastern Roe Deer	Blue Eared Pheasant	0.64	0.58–0.69	0	0.71	0.67–0.75	0
Eastern Roe Deer	Wild Boar	0.82	0.72–0.9	0.04	0.86	0.77–0.92	0.31
Eastern Roe Deer	Tolai Hare	0.89	0.82–0.95	0.34	0.72	0.62–0.78	0
Eastern Roe Deer	Asian Badger	0.85	0.77–0.9	0.01	0.77	0.64–0.87	0.24
Blue Eared Pheasant	Blood Pheasant	0.73	0.62–0.85	0	0.87	0.78–0.93	0.24
Blue Eared Pheasant	Tolai Hare	0.64	0.56–0.73	0	0.46	0.36–0.54	0
Blue Eared Pheasant	Common Pheasant	0.8	0.68–0.89	0.06	0.88	0.79–0.94	0.24
Wild Boar	Tolai Hare	0.75	0.63–0.86	0	0.67	0.54–0.78	0
Wild Boar	Asian Badger	0.78	0.67–0.87	0	0.72	0.57–0.85	0.02
Wild Boar	Red Fox	0.75	0.6–0.85	0.01	0.76	0.63–0.86	0
Blood Pheasant	Tolai Hare	0.66	0.54–0.76	0	0.39	0.3–0.47	0
Blood Pheasant	Common Pheasant	0.58	0.43–0.73	0	0.86	0.78–0.92	0.22
Tolai Hare	Common Pheasant	0.6	0.46–0.74	0	0.48	0.38–0.57	0
Tolai Hare	Asian Badger	0.87	0.79–0.93	0.17	0.8	0.59–0.91	0.17
Asian Badger	Red Fox	0.9	0.83–0.95	0.6	0.84	0.72–0.93	0.71

**Table 5:** Coefficients of daily activity overlapping (Dhat4), confidence intervals (CI) and *p*-level (*p*) for species inside and outside the reserve on the eastern Qinghai-Tibet Plateau in 2017–2020.

		Inside			Outside		
Species1	Species2	Dhat4	CI	*p*	Dhat4	CI	*p*
Eastern Roe Deer	Blue Eared Pheasant	0.65	0.61–0.68	0	0.7	0.56–0.8	0.01
Eastern Roe Deer	Wild Boar	0.85	0.79–0.91	0	0.78	0.63–0.89	0.13
Eastern Roe Deer	Asian Badger	0.86	0.77–0.92	0.02	0.89	0.81–0.94	1
Blue Eared Pheasant	Wild Boar	0.54	0.45–0.6	0	0.8	0.67–0.91	0.42
Blue Eared Pheasant	Asian Badger	0.58	0.48–0.67	0	0.66	0.53–0.8	0.01
Wild Boar	Asian Badger	0.82	0.74–0.9	0.03	0.8	0.67–0.91	0.06

Statistical significance of Watson’s two-sample test: *p* value < 0.05 means significant difference.

## Data Availability

The datasets used in this study are available from the corresponding author on reasonable request.

## Appendix A. Information on Camera Sites on the Eastern Qinghai-Tibet Plateau

**Table:**

Site ID	Site Name	Elevation	Reserve Part
A01	F22	2800	inside
A02	Ftouling	2843	inside
A03	qingyatou	2924	inside
A04	shuihutan	2749	inside
A05	B39	2873	inside
A06	heiniuheliang	2904	inside
A07	taihuangpoliang	3103	inside
A08	yayatan	3120	inside
A09	xuegouliang	3106	inside
A10	tushanliang	3146	inside
A11	gangouliang	2967	inside
A12	gonggouwan	2736	inside
A13	dahegou	2802	inside
A14	mamianzui	2595	inside
A15	shiwaliang	3059	inside
A16	macheshanliang	3168	inside
A17	macheshanliang2	3391	inside
A18	macheshan3	3468	inside
A19	gangouliangwai	2821	inside
A20	sxiantou	2953	inside
B01	shanzhuangliang	2384	inside
B02	majuanliang	2550	inside
B03	majuangouli	2441	inside
B04	majuangouzhong	2350	inside
B05	donggoutan	2457	inside
B06	daochaxia	2583	inside
B07	daochashang	2592	inside
B08	jishanliang	2570	inside
B09	baogoukou	2589	inside
B10	zhuojitan	2148	inside
B11	shagugoukou	2132	inside
B12	zuguchuanshalukou	2097	inside
B13	shelugou	2423	inside
B14	gabodanwan	2299	inside
B15	gougushan	2399	inside
B16	anzanggou	2192	inside
B17	maoergou	2251	inside
B18	wowode	2324	inside
B19	gulonggou	2148	inside
B20	yazakan	2093	inside
C01	heihegou01	2825	out
C02	heihegou02	2782	out
C03	heihegou03	2735	out
C04	heihegou04	2653	out
C05	heihegou05	2704	out
C06	heihegou06	2603	out
C07	heihegou07	2503	out
C08	xialijiatieqiao	2378	out
C09	taihe09	2394	out
C10	taihe10	2367	out
C11	taihe11	3144	out
C12	taihe12	3106	out
C13	taihe13	3012	out
C14	taihe14	2899	out
C15	taihe15	2815	out
C16	taihe16	2696	out
C17	taihe17	2806	out
C18	taihe18	2500	out
C19	taihe19	2527	out
C20	taihe20	2498	out
